# Genetic polymorphisms are associated with the risk of gastric and colorectal cancers in a Han Chinese population

**DOI:** 10.18632/oncotarget.15745

**Published:** 2017-02-26

**Authors:** Nan Wang, Qing Qiao, Guoqiang Bao, Tao Wu, Yizhou Li, Jingjie Li, Jianguo Lu, Xianli He

**Affiliations:** ^1^ Department of General Surgery, Tangdu Hospital, Fourth Military Medical University, Xi’an, Shaanxi, 710038, China; ^2^ Inner Mongolia Medical University, Hohhot, Inner Mongolia, 010050, China; ^3^ Key Laboratory of Resource Biology and Biotechnology in Western China, Ministry of Education, School of Life Sciences, Northwest University, Xi’an, Shaanxi, 710069, China

**Keywords:** gastric cancer, colorectal cancer, single-nucleotide polymorphism, susceptibility, case-control

## Abstract

Here, we genotyped eleven single-nucleotide polymorphisms (SNPs) and evaluated their association with the risk of developing gastric cancer (GC) or colorectal cancer (CRC) in 1,790 Han Chinese participants (588 GC patients, 499 CRC patients, and 703 healthy controls). Statistically analysis showed that the “C” allele of rs2689154 in *MIPEPP2* was associated with a decreased risk of GC (odds ratio [OR] = 0.81, 95 % confidence interval [CI]: 0.66-0.99, *P* = 0.041), whereas the “T” allele of rs12615966 in *LOC284998* was associated with a 1.29-fold increase in the risk of GC (OR = 1.29, 95% CI: 1.03-1.63, *P* = 0.029). Additionally, genetic model analyses showed that rs2689154 was associated with a reduced risk of GC under the recessive model (adjusted OR = 0.46, 95% CI: 0.22-0.98, *P* = 0.037), and rs12615966 in *FOXF1* was associated with an increased risk of GC in both the dominant and log-additive models after adjusted for age and gender (adjusted OR = 1.36, 95% CI: 1.02-1.81, *P* = 0.033; adjusted OR = 1.36, 95% CI: 1.05-1.75, *P* = 0.018, respectively). We also observed that rs2178146 in *FOXF1* was associated with an increased risk of CRC in the recessive model (adjusted OR = 1.90, 95% CI: 1.05-3.45, *P* = 0.034). Our results confirmed that rs2689154 in *MIPEPP2* was significantly decreased GC risk, but rs12615966 in *LOC284998* was significantly increased GC risk, and rs2178146 in *FOXF1* was associated with increased CRC risk in the Han Chinese population.

## INTRODUCTION

Gastric cancer (GC) is a major public health problem worldwide and accounts for a notable proportion of global cancer mortality [1]. An estimated 951,600 new cases of and 723,100 deaths due to stomach cancer occurred in 2012, and GC incidence rates are highest in Eastern Asia (particularly in Korea, Japan, and China) [2]. Although the mechanisms underlying GC pathogenesis remain unclear, epidemiological studies have identified some environmental risk factors, including smoking, alcohol consumption, dietary deficiencies, gastroesophageal reflux [3], and *Helicobacter pylori* infection [4]. However, only a small proportion of individuals exposed to these risk factors actually develop GC, suggesting that genetic factors also play a vital role in susceptibility to GC.

Colorectal cancer (CRC) is the third most commonly diagnosed cancer in males and the second in females, with an estimated 1.4 million cases of and 693,900 deaths due to CRC occurring in 2012 [2]. Epidemiological studies have demonstrated that environmental factors, including smoking, alcohol consumption, dietary patterns, obesity, and physical inactivity were associated with the risk of developing CRC [5]. Genetic factors have also been established as important contributors to CRC etiology [6]. Large-scale genome-wide association studies (GWASs) have identified numerous single nucleotide polymorphisms (SNPs) that are associated with susceptibility to CRC [7, 8]. A better understanding of the genetic factors that contribute to CRC might help identify the mechanisms underlying CRC pathogenesis.

Genome-wide association studies (GWAS) have demonstrated that rs2689154 (*MIPEPP2*), rs4927850 (*LOC105374300*), rs2255280 (*DAB2*), rs12615966 (*LOC284998*), rs7574865 (*STAT4*), and rs3790844 (*NR5A2*) SNPs are associated with an increased risk of pancreatic cancer in Japanese [9, 10] and Chinese population [11]. Additionally, some reports have shown that rs12100561 (*C14orf143*), rs2178146 (*FOXF1*), and rs1050631 (*SLC39A6*) are associated with increased susceptibility to hepatocellular carcinoma [12], esophageal adenocarcinoma [13], and esophageal squamous-cell carcinoma [14], respectively. However, to our knowledge, few studies have examined associations between polymorphisms and the risk of GC or CRC. For example, although the *SPARCL1* gene is associated with the risk of GC [15] and CRC [16], the association between rs4610302 and susceptibility to GC and CRC in the Han Chinese population has not yet been examined. In addition, The SNP rs4591517 (*SALL4P5-RPL24P7*) has been associated with the risk of CRC, but the association between this SNP and the risk of GC remains unknown.

In this case-control study, we investigated whether eleven SNPs (rs3790844, rs2689154, rs12615966, rs7574865, rs4591517, rs4927850, rs4610302, rs2255280, rs12100561, rs2178146, and rs1050631) were associated with susceptibility to GC and CRC in a Han Chinese population.

## RESULTS

Basic patient characteristics for all subjects are listed in Table 1. A total of 588 GC patients (392 males and 196 females) with a mean age of 58.1 (±11.7) years, 499 CRC patients (260 males and 189 females) with a mean age of 59.1 (±11.8) years, and 703 healthy controls (396 males and 307 females) with a mean age of 48.6 (±9.4) years were enrolled in our study. GC patients and healthy controls differed regarding age and sex (*P* < 0.001) (Table 1), while CRC patients and healthy controls differed in age (*P* < 0.001), but not sex (*P* = 0.598). In order to eliminate residual confounding effects associated with these differences, subsequent multivariate unconditional logistic regression analyses were adjusted for age and gender.

**Table 1:** Characteristics of cancer patients and healthy controls

Characteristic	Case (N=588)	GC	*P*-value	Case (N=449)	CRC	*P*-value
		Control (N=703)			Control (N=703)	
Gender			**< 0.001**			0.598
Male (%)	392 (66.7)	396 (56.3)		260 (57.9)	396 (56.3)	
Female (%)	196 (33.3)	307 (43.7)		189 (42.1)	307 (43.7)	
Age			**< 0.001**			**< 0.001**
Mean age ± SD	58.1 ± 11.7	48.6 ± 9.4		59.1 ± 11.8	48.6 ± 9.4	

GC: Gastric cancer; CRC: Colorectal cancer.

*P* < 0.05 indicates statistical significance.

The allele distributions and minor allele frequencies (MAF) for each SNP, and the results of the Hardy-Weinberg equilibrium (HWE) test, are shown in Table 2. All eleven SNPs were in HWE in control subjects (*P* > 0.05) (Table 2). Differences in allele frequency distributions between cancer patients and healthy controls were identified using Chi-squared tests; two SNPs were associated with susceptibility to GC (Table 2). The “C” allele of rs2689154 in *MIPEPP2* was associated with a decreased risk of GC (OR = 0.81, 95 % CI: 0.66-0.99, *P* = 0.041), while the “T” allele of rs12615966 in *LOC284998* was associated with a 1.29-fold increase in the risk of GC (OR = 1.29, 95% CI: 1.03-1.63, *P* = 0.029).

**Table 2:** Association analysis of SNP allele frequencies in cancer patients and controls

SNP-ID	Gene(s)	Chromosome	Allele A/B	MAF			HWE *P*-value	OR	GC	*P*-value	OR	CRC	*P*-value
				Case^1^	Case^2^	Control			95% CI			95% CI	
rs3790844	*NR5A2*	1q32.1	T/C	0.272	0.309	0.306	0.131	0.845	0.712-1.003	0.054	1.014	0.853-1.215	0.884
rs2689154	*MIPEPP2*	1q43	C/G	0.168	0.183	0.199	0.906	0.811	0.663-0.991	**0.041**	0.899	0.725-1.113	0.327
rs12615966	*LOC284998*	2q12.1	T/C	0.145	0.122	0.116	0.853	1.293	1.027-1.629	**0.029**	1.068	0.825-1.383	0.615
rs7574865	*STAT4*	2q32.3	T/G	0.336	0.339	0.341	0.675	0.979	0.831-1.153	0.799	0.989	0.829-1.180	0.905
rs4591517	*SALL4P5 - RPL24P7*	3p24.3	T/C	0.202	0.174	0.185	0.615	1.112	0.913-1.354	0.291	0.927	0.744-1.153	0.496
rs4927850	*LOC105374300*	3q29	T/C	0.207	0.184	0.196	0.905	1.071	0.883-1.299	0.485	0.926	0.748-1.148	0.486
rs4610302	*SPARCL1*	4q22.1	A/G	0.395	0.364	0.373	0.809	1.099	0.937-1.289	0.245	0.962	0.808-1.145	0.664
rs2255280	*DAB2*	5p13.1	C/A	0.336	0.342	0.333	0.061	1.015	0.861-1.196	0.861	1.041	0.872-1.242	0.660
rs12100561	*C14orf143*	14q32.11	A/G	0.417	0.398	0.424	0.088	0.971	0.830-1.137	0.718	0.898	0.757-1.065	0.216
rs2178146	*FOXF1*	16q24.1	G/A	0.226	0.248	0.222	0.101	1.025	0.851-1.234	0.795	1.158	0.951-1.410	0.143
rs1050631	*SLC39A6*	18q12.2	T/C	0.160	0.166	0.159	0.674	1.006	0.814-1.243	0.955	1.05	0.837-1.317	0.675

MAF: Minor allele frequency; HWE: Hardy-Weinberg equilibrium; OR: Odds ratio; 95% CI: 95% Confidence interval.

Case^1^ refers to gastric cancer case; Case^2^ refers to colorectal cancer case.

*P* < 0.05 indicates statistical significance.

Unconditional logistic regression analysis was then used to evaluate different genetic models (codominant, dominant, recessive, overdominant, and log-additive) for the eleven SNPs (Table 3). The rs2689154 SNP in *MIPEPP2* was associated with a reduced risk of GC in both the recessive model after adjusted for age and gender (adjusted OR = 0.46, 95% CI: 0.22-0.98, *P* = 0.037) and the log-additive model without adjustment (OR = 0.81, 95% CI: 0.66-0.99, *P* = 0.038). In contrast, the rs12615966 SNP in *LOC284998* was associated with an increased risk of GC in both the dominant model (adjusted OR = 1.36, 95% CI: 1.02-1.81, *P* = 0.033) and the log-additive model (adjusted OR = 1.36, 95% CI: 1.05-1.75, *P* = 0.018) after adjusted for age and gender. Finally, rs2178146 in *FOXF1* was associated with an increased risk of CRC in the recessive model both before and after adjustment for age and gender (OR = 2.05, 95% CI: 1.22-3.45, *P* = 0.007; adjusted OR =1.90, 95% CI: 1.05-3.45, *P* = 0.034).

**Table 3:** Genetic model analyses of associations between SNPs and cancer risk

Cancer	SNP-ID	Model	Genotype	Control	Case	Without adjustment		With adjustment	
						OR (95% CI)	*P*-value	OR (95% CI)	*P*-value
GC	rs2689154	Codominant	G/G	450 (64%)	402 (68.5%)	1.00	0.073	1.00	0.096
			G/C	226 (32.1%)	173 (29.5%)	0.86 (0.67-1.09)		0.92 (0.71-1.20)	
			C/C	27 (3.8%)	12 (2%)	0.50 (0.25-1.00)		0.45 (0.21-0.96)	
		Dominant	G/G	450 (64%)	402 (68.5%)	1.00	0.091	1.00	0.280
			G/C-C/C	253 (36%)	185 (31.5%)	0.82 (0.65-1.03)		0.87 (0.67-1.12)	
		Recessive	G/G-G/C	676 (96.2%)	575 (98%)	1.00	0.056	1.00	**0.037**
			C/C	27 (3.8%)	12 (2%)	0.52 (0.26-1.04)		0.46 (0.22-0.98)	
		Overdominant	G/G-C/C	477 (67.8%)	414 (70.5%)	1.00	0.300	1.00	0.730
			G/C	226 (32.1%)	173 (29.5%)	0.88 (0.70-1.12)		0.95 (0.73-1.24)	
		Log-additive	---	---	---	0.81 (0.66-0.99)	**0.038**	0.83 (0.67-1.04)	0.110
	rs12615966	Codominant	C/C	549 (78.3%)	434 (73.8%)	1.00	0.081	1.00	0.054
			C/T	142 (20.3%)	138 (23.5%)	1.23 (0.94-1.60)		1.31 (0.97-1.75)	
			T/T	10 (1.4%)	16 (2.7%)	2.02 (0.91-4.50)		2.23 (0.91-5.47)	
		Dominant	C/C	549 (78.3%)	434 (73.8%)	1.00	0.058	1.00	**0.033**
			C/T-T/T	152 (21.7%)	154 (26.2%)	1.28 (0.99-1.66)		1.36 (1.02-1.81)	
		Recessive	C/C-C/T	691 (98.6%)	572 (97.3%)	1.00	0.100	1.00	0.100
			T/T	10 (1.4%)	16 (2.7%)	1.93 (0.87-4.29)		2.10 (0.86-5.14)	
		Overdominant	C/C-T/T	559 (79.7%)	450 (76.5%)	1.00	0.160	1.00	0.100
			C/T	142 (20.3%)	138 (23.5%)	1.21 (0.93-1.57)		1.28 (0.95-1.72)	
		Log-additive	---	---	---	1.28 (1.02-1.61)	**0.031**	1.36 (1.05-1.75)	**0.018**
CRC	rs2178146	Codominant	A/A	418 (59.5%)	260 (57.9%)	1.00	**0.024**	1.00	0.086
			G/A	258 (36.7%)	155 (34.5%)	0.97 (0.75-1.24)		1.10 (0.83-1.46)	
			G/G	27 (3.8%)	34 (7.6%)	2.02 (1.19-3.43)		1.97 (1.07-3.61)	
		Dominant	A/A	418 (59.5%)	260 (57.9%)	1.00	0.600	1.00	0.220
			G/A-G/G	285 (40.5%)	189 (42.1%)	1.07 (0.84-1.36)		1.18 (0.90-1.55)	
		Recessive	A/A-G/A	676 (96.2%)	415 (92.4%)	1.00	**0.007**	1.00	**0.034**
			G/G	27 (3.8%)	34 (7.6%)	2.05 (1.22-3.45)		1.90 (1.05-3.45)	
		Overdominant	A/A-G/G	445 (63.3%)	294 (65.5%)	1.00	0.450	1.00	0.780
			G/A	258 (36.7%)	155 (34.5%)	0.91 (0.71-1.16)		1.04 (0.79-1.37)	
		Log-additive	---	---	---	1.16 (0.95-1.41)	0.140	1.23 (0.98-1.54)	0.070

GC: Gastric cancer; CRC: Colorectal cancer; OR: Odds ratio; 95% CI: 95% Confidence interval.

*P* values were calculated from unconditional logistic regression analysis.

*P* < 0.05 indicates statistical significance.

## DISCUSSION

In this case-control study, we investigated whether eleven SNPs associated with pancreatic cancer, esophageal cancer, CRC, and other digestive system cancers were associated with susceptibility to GC and CRC in a Han Chinese population. We found that the rs2689154 SNP in *MIPEPP2* was protective against GC. In contrast, the rs12615966 SNP in *LOC284998* was associated with an increased risk of GC. In addition, the rs2178146 SNP in *FOXF1* was associated with an increased risk of CRC.

Previous genome-wide association studies demonstrated that the rs2689154 SNP in *MIPEPP2* and the rs12615966 SNP in *LOC284998* are associated with the risk of developing pancreatic cancer in Chinese [11] and Japanese [9] populations, respectively. However, associations between these two SNPs and the risk of developing other types of cancer have not been examined. Here, we found that rs2689154 was protective against GC, while rs12615966 was associated with an increased risk of GC, in a Han Chinese patient population; additional studies should be conducted with larger sample sizes and in other populations to confirm these findings.

In a previous study, the SNP rs2178146 in *FOXF1*, a member of the forkhead family of transcription factors, was associated with susceptibility to esophageal adenocarcinoma [13]. *FOXF1*, a potential tumor suppressor gene that is epigenetically silenced in breast cancer, plays a critical role in embryonic development as well as in cell cycle processes that maintain genomic stability [17, 18]. In addition, *FOXF1* is a novel gene target of the p53 family, and ectopic expression and inactivation of *FOXF1* inhibited and stimulated, respectively, cancer cell invasion and migration [19]. Furthermore, *FOXF1* expression is largely silenced in colorectal cancer cell lines with inactive p53, and knockdown of *FOXF1* caused genomic instability in colorectal cancer cells with a defect in the p53-p21^WAF1^ checkpoint, suggesting that *FOXF1* plays an essential role in colorectal tumorigenesis [20]. Mesenchymal stem cells (MSCs) are also involved in colorectal tumor development and progression [21, 22]. Interestingly, *FOXF1* contributes to the anti-malignant effects of the fusion of MSCs with cancer cells by regulating the expression of p21 [23]. In addition, abnormal activation of the Hedgehog (Hh) signaling pathway, which also plays an important role in human development, has been observed in several types of human cancers, including GC [24] and CRC [25]. Furthermore, Hh signals indirectly up-regulate BMP4 levels via *FOXF1* to induce vascular tube formation [26]. It is therefore plausible that the rs2178146 SNP in *FOXF1* may increase the risk of developing CRC by decreasing the anti-malignant effects of MSC fusion, influencing Hedgehog signaling, or inhibiting vascular tube formation.

Some potential limitations of the current study should be considered when interpreting the results. First, the sample size was relatively small. Second, many other risk factors (e.g., smoking, alcohol consumption) were not examined due to the lack of relevant clinical data. Third, the biological functions of these SNPs were not analyzed and should be investigated in future studies. In addition, the novel associations identified here should be confirmed in additional studies with larger sample sizes.

In conclusion, we found that the rs2689154 SNP in *MIPEPP2* and the rs12615966 SNP in *LOC284998* were associated with susceptibility to GC, while the rs2178146 SNP in *FOXF1* was associated with an increased risk of CRC, in a Han Chinese population. Our findings can provide a theoretical foundation for further researcher, and the associations between these SNPs and the risk of developing GC and CRC should be examined in other populations or with larger samples.

## MATERIALS AND METHODS

### Ethics statement

The study protocol was approved by the Ethics Committee of the Tangdu Hospital, Fourth Military Medical University, and complied with the Declaration of Helsinki. All individuals gave written informed consent prior to participation in the study.

### Study participants

A total of 1,790 participants (588 GC patients, 499 CRC patients, and 703 healthy individuals) were included in the study. All cases were recruited from the Tangdu Hospital, Fourth Military Medical University between January 2011 and February 2014. The healthy controls were randomly selected from the health examination ward of Tangdu Hospital during the same period. Inclusion and exclusion criteria were as follows: all subjects were unrelated ethnic Han Chinese whose ancestors had lived in the region for at least the three generations; all included cases were recently diagnosed by histopathological confirmation according to the criteria established by the Union for International Cancer Control tumor-node-metastasis (TNM) classification system; patients with personal or family histories of inflammatory or autoimmune diseases in the intestine, other cancers, chemotherapy, or radiotherapy were excluded; patients were chosen without restrictions regarding age, gender, or disease stage. Basic characteristics for all enrolled controls were collected by well-trained interviewers using standard epidemiological questionnaires. Case information for cancer patients was collected through consultations with treating physicians or by reviewing medical charts.

### DNA extraction

Peripheral venous blood (5 mL) was collected from each participant using vacutainer tubes containing ethylene diamine tetra-acetic acid (EDTA) and then stored at −80°C. Genomic DNA was extracted from whole-blood samples using the GoldMag-Mini Whole Blood Genomic DNA Purification Kit according to the manufacturer's protocol (GoldMag. Co. Ltd., Xi’an, China). The DNA samples were preserved at −4°C for future use. DNA concentrations were evaluated by measuring absorbance at 260nm and 280nm using a spectrophotometer (NanoDrop 2000; Thermo Fisher Scientific, Waltham, MA, USA). DNA was quantified and diluted using QIAgility to a final concentration of 20 ng/μL.

### Genotyping

The eleven SNPs (rs3790844, rs2689154, rs12615966, rs7574865, rs4591517, rs4927850, rs4610302, rs2255280, rs12100561, rs2178146, and rs1050631) associated with pancreatic cancer, esophageal cancer, and other digestive system cancers were randomly chosen from previous GWAS reports for examination [9–11, 13, 14]. The minor allele frequencies for all of the SNPs were > 5% for the Chinese Han Beijing (CHB) population in HapMap. SNP genotyping was performed using the Sequenom MassARRAY platform (Sequenom, San Diego, CA, USA) according to the standard protocol recommended by the manufacturer. Sequenom Typer 4.0 software was used for data management and analyses.

### Statistical analysis

SPSS18.0 statistical software (SPSS Inc., Chicago, IL, USA) and Microsoft Excel (Microsoft Corp., Redmond, WA, USA) were used for statistical analysis. The age distributions of the cancer patients and healthy controls were compared using Welch's *t*-test, and the gender distributions were compared using chi-square tests. Pearson's test was used to assess the variation in each SNP frequency from the Hardy-Weinberg equilibrium (HWE) in the control subjects. The allele frequencies for the two groups were compared with a chi-square test, and the relative risk was estimated using odds ratios (ORs) and 95% confidence intervals (CIs). The associations between SNPs and GC and CRC were tested in genetic models (codominant, dominant, recessive, overdominant, and log-additive models), and associated ORs and 95% CIs were determined using unconditional logistic regression analysis with adjustments for age and gender. All *P* values presented in this study are two sided; *P* < 0.05 was considered statistically significant.

## References

[1] McLean MH, El-Omar EM (2014). Genetics of gastric cancer. Nat Rev Gastroenterol Hepatol.

[2] Torre LA, Bray F, Siegel RL, Ferlay J, Lortet-Tieulent J, Jemal A (2015). Global cancer statistics, 2012. CA Cancer J Clin.

[3] Mayne ST, Navarro SA (2002). Diet, obesity and reflux in the etiology of adenocarcinomas of the esophagus and gastric cardia in humans. J Nutr.

[4] The EUROGAST Study Group (1993). An international association between Helicobacter pylori infection and gastric cancer. Lancet.

[5] Haggar FA, Boushey RP (2009). Colorectal cancer epidemiology: incidence, mortality, survival, and risk factors. Clin Colon Rectal Surg.

[6] de la Chapelle A (2004). Genetic predisposition to colorectal cancer. Nat Rev Cancer.

[7] Houlston RS, Cheadle J, Dobbins SE, Tenesa A, Jones AM, Howarth K, Spain SL, Broderick P, Domingo E, Farrington S, Prendergast JG, Pittman AM, Theodoratou E, COGENT Consortium, and CORGI Consortium, and COIN Collaborative Group, and COINB Collaborative Group (2010). Meta-analysis of three genome-wide association studies identifies susceptibility loci for colorectal cancer at 1q41, 3q26.2, 12q13.13 and 20q13.33. Nat Genet.

[8] Zhang B, Jia WH, Matsuda K, Kweon SS, Matsuo K, Xiang YB, Shin A, Jee SH, Kim DH, Cai Q, Long J, Shi J, Wen W (2014). Large-scale genetic study in East Asians identifies six new loci associated with colorectal cancer risk. Nat Genet.

[9] Low SK, Kuchiba A, Zembutsu H, Saito A, Takahashi A, Kubo M, Daigo Y, Kamatani N, Chiku S, Totsuka H, Ohnami S, Hirose H, Shimada K (2010). Genome-wide association study of pancreatic cancer in Japanese population. PLoS One.

[10] Ueno M, Ohkawa S, Morimoto M, Ishii H, Matsuyama M, Kuruma S, Egawa N, Nakao H, Mori M, Matsuo K, Hosono S, Nojima M, Wakai K (2015). Genome-wide association study-identified SNPs (rs3790844, rs3790843) in the NR5A2 gene and risk of pancreatic cancer in Japanese. Sci Rep.

[11] Wu C, Miao X, Huang L, Che X, Jiang G, Yu D, Yang X, Cao G, Hu Z, Zhou Y, Zuo C, Wang C, Zhang X (2011). Genome-wide association study identifies five loci associated with susceptibility to pancreatic cancer in Chinese populations. Nat Genet.

[12] Clifford RJ, Zhang J, Meerzaman DM, Lyu MS, Hu Y, Cultraro CM, Finney RP, Kelley JM, Efroni S, Greenblum SI, Nguyen CV, Rowe WL, Sharma S (2010). Genetic variations at loci involved in the immune response are risk factors for hepatocellular carcinoma. Hepatology.

[13] Becker J, May A, Gerges C, Anders M, Veits L, Weise K, Czamara D, Lyros O, Manner H, Terheggen G, Venerito M, Noder T, Mayershofer R (2015). Supportive evidence for FOXP1, BARX1, and FOXF1 as genetic risk loci for the development of esophageal adenocarcinoma. Cancer Med.

[14] Wu C, Li D, Jia W, Hu Z, Zhou Y, Yu D, Tong T, Wang M, Lin D, Qiao Y, Zhou Y, Chang J, Zhai K (2013). Genome-wide association study identifies common variants in SLC39A6 associated with length of survival in esophageal squamous-cell carcinoma. Nat Genet.

[15] Li P, Qian J, Yu G, Chen Y, Liu K, Li J, Wang J (2012). Down-regulated SPARCL1 is associated with clinical significance in human gastric cancer. J Surg Oncol.

[16] Zhang H, Widegren E, Wang DW, Sun XF (2011). SPARCL1: a potential molecule associated with tumor diagnosis, progression and prognosis of colorectal cancer. Tumour biology.

[17] Lo PK, Lee JS, Liang X, Han L, Mori T, Fackler MJ, Sadik H, Argani P, Pandita TK, Sukumar S (2010). Epigenetic inactivation of the potential tumor suppressor gene FOXF1 in breast cancer. Cancer Res.

[18] Ren X, Ustiyan V, Pradhan A, Cai Y, Havrilak JA, Bolte CS, Shannon JM, Kalin TV, Kalinichenko VV (2014). FOXF1 transcription factor is required for formation of embryonic vasculature by regulating VEGF signaling in endothelial cells. Circ Res.

[19] Tamura M, Sasaki Y, Koyama R, Takeda K, Idogawa M, Tokino T (2014). Forkhead transcription factor FOXF1 is a novel target gene of the p53 family and regulates cancer cell migration and invasiveness. Oncogene.

[20] Lo PK, Lee JS, Sukumar S (2012). The p53-p21WAF1 checkpoint pathway plays a protective role in preventing DNA rereplication induced by abrogation of FOXF1 function. Cell Signal.

[21] Shinagawa K, Kitadai Y, Tanaka M, Sumida T, Kodama M, Higashi Y, Tanaka S, Yasui W, Chayama K (2010). Mesenchymal stem cells enhance growth and metastasis of colon cancer. Int J Cancer.

[22] De Boeck A, Pauwels P, Hensen K, Rummens JL, Westbroek W, Hendrix A, Maynard D, Denys H, Lambein K, Braems G, Gespach C, Bracke M, De Wever O (2013). Bone marrow-derived mesenchymal stem cells promote colorectal cancer progression through paracrine neuregulin 1/HER3 signalling. Gut.

[23] Wei HJ, Nickoloff JA, Chen WH, Liu HY, Lo WC, Chang YT, Yang PC, Wu CW, Williams DF, Gelovani JG, Deng WP (2014). FOXF1 mediates mesenchymal stem cell fusion-induced reprogramming of lung cancer cells. Oncotarget.

[24] Katoh Y, Katoh M (2005). Hedgehog signaling pathway and gastric cancer. Cancer Biol Ther.

[25] Yoshikawa K, Shimada M, Miyamoto H, Higashijima J, Miyatani T, Nishioka M, Kurita N, Iwata T, Uehara H (2009). Sonic hedgehog relates to colorectal carcinogenesis. J Gastroenterol.

[26] Astorga J, Carlsson P (2007). Hedgehog induction of murine vasculogenesis is mediated by Foxf1 and Bmp4. Development.

